# CDK5RAP3 Inhibits the Translocation of MCM6 to Influence the Prognosis in Gastric Cancer

**DOI:** 10.7150/jca.32208

**Published:** 2019-07-25

**Authors:** Qi-Yue Chen, Li-Chao Liu, Jia-Bin Wang, Jian-Wei Xie, Jian-Xian Lin, Jun Lu, Long-Long Cao, Mi Lin, Ru-Hong Tu, Chang-Ming Huang, Ping Li, Chao-Hui Zheng

**Affiliations:** 1 Department of Gastric Surgery, Fujian Medical University Union Hospital, Fuzhou, China; 2 Department of General Surgery, Fujian Medical University Union Hospital, Fuzhou, China; 3 Key Laboratory of Ministry of Education of Gastrointestinal Cancer, Fujian Medical University, Fuzhou, China; 4 Fujian Key Laboratory of Tumor Microbiology, Fujian Medical University, Fuzhou, China

**Keywords:** CDK5RAP3, MCM6, Gastric cancer, Survival

## Abstract

Cyclin-dependent kinase 5 regulatory subunit-associated protein 3 (CDK5RAP3) was identified as a tumor suppressor in gastric cancer, while, minichromosome maintenance complex component 6 (MCM6), which is closely related to the initiation of DNA replication, was reported to be upregulated in multiple malignancies. However, the interaction between these two proteins has not been investigated in gastric cancer. Here, we evaluate the connection between CDK5RAP3 and MCM6 using mass spectrometry and immunoprecipitation. In cells, cell growth and invasiveness indicate that CDK5RAP3 acts as a tumor suppressor by preventing the effects of MCM6. The potential mechanism was revealed using immunofluorescence and nuclear protein extraction. In patients, immunohistochemistry and immunofluorescence show that the protein levels of CDK5RAP3 were markedly decreased in most gastric tumor tissues compared with adjacent nontumor tissues, and the expression levels of MCM6 in the nucleus showed the opposite trend. Prognostic analysis showed that the combined expression of CDK5RAP3 and MCM6 was an independent prognostic factor correlating with the overall survival of gastric cancer patients. Cox regression analysis indicated that the expression of CDK5RAP3 and MCM6 corresponded to T, N, and M stages. Our results demonstrate that CDK5RAP3 can interact with MCM6 and prevent MCM6 from translocating into the nucleus, which may be a potential mechanism through which CDK5RAP3 negatively regulates the proliferation of gastric cancer.

## Introduction

Gastric cancer (GC) is the fourth most common cancer in the world with more than 70% of cases occurring in the developing world, especially in Eastern Asia 1. Figuring out the mechanism involved in gastric cancer will help to evaluate patients' prognoses, and to develop novel therapies. The cyclin-dependent kinase 5 regulatory subunit-associated protein 3 (CDK5RAP3, also called C53/LZAP) was originally identified as a binding partner of the CDK5 activator p35 using the yeast two-hybrid system 2. Northern blot analysis indicates that the expression levels of CDK5RAP3 are relatively constant in the heart, brain, lung, liver, kidney and pancreas 3. Our previous study found that the downregulation of CDK5RAP3 leads to poor prognosis in gastric cancer via inhibition of Wnt/β-catenin signaling 4. While there have been many other proteins that can interact with CDK5RAP3, the results of mass spectrometry (MS) suggested that members of the family of minichromosome maintenance proteins (MCMs) may have an interaction with CDK5RAP3.

MCMs comprise a group of proteins that is closely related to DNA replication initiators, including MCM2-9 5. In the initiation of DNA replication licensing process, MCM2-7 form a complex that combines chromatin for to create the origins of DNA replication during the late M to early G1 phase of the cell cycle 6. It has been reported that the elevation of MCM expression contributes to cell proliferation and tumorigenesis. For example, MCM2, MCM5, MCM6 and MCM7 have been reported to be upregulated in cervical epithelium squamous cancers, renal cell carcinomas, hepatocellular carcinomas and esophageal squamous cell carcinomas, leading to poorer prognosis 7-10. These studies suggest that MCMs play an important role in tumorigenesis and proliferation.

Some studies have indicated that MCM6, a member of the MCMs family, is upregulated in various types of malignancies. For example, in meningiomas, MCM6 expression levels are higher in recurrent tumors than in indolent tumors 11. In non-small cell lung carcinoma, MCM6 expression was upregulated as well 12, while in gastric cancer, there have been few studies to determine the potential involvement of MCM6.

In this study, we performed a series of experiments to explore the interaction between CDK5RAP3 and MCM6 and evaluated the prognostic value of their combined expression. We discovered that CDK5RAP3 could interact with MCM6 and prevent it from translocating into the nucleus in gastric cancer.

## Materials and Methods

### Human gastric tumor tissues

The human gastric tumor tissues of 206 patients were obtained from Fujian Medical University Union Hospital (Fujian, China) with detailed clinical-pathological parameters. 136 patients for TMA underwent radical gastrectomy from 2013 to 2015 and 70 patients for an independent cohort underwent radical gastrectomy from 2011 to 2014. The inclusion criteria were as follows: (1) patients who had a histological confirmation of SRC or tubular adenocarcinoma; (2) >15 lymph nodes were retrieved; (3) patients who underwent R0 resection. The following exclusion criteria were used: (1) multiple primary tumors; (2) neoadjuvant chemotherapy was administered; (3) remnant gastric cancer was found; and (4) incomplete pathological data. The pathological stage of the tumor was reassessed according to the 2010 International Union Against Cancer (UICC) TNM classification on gastric cancer (seventh edition) 13. The respective adjacent nontumor tissues were located at least 5 cm from the gastric tumor. The 136 paraffin-embedded gastric tumor and respective adjacent nontumor tissues were collected for immunohistochemistry (IHC) from 2013 to 2015. This study was approved by the ethics committee of Fujian Medical University Union Hospital, and written consent was obtained from all patients involved.

### Follow-up

Trained doctors systematically followed up with all patients based on an institutional follow-up protocol using several approaches including outpatient service, letters, telephone, email or visits. Follow-up was conducted every 3 months during the first year, every 6 months beyond the second year, and all surviving patients will be followed for more than three years. The survival time was the time from the date of surgery until the last contact, or the date of death.

### Tissue microarray (TMA)

A series of TMAs containing gastric cancer samples were constructed. Briefly, all the gastric cancer tissues were reviewed by a pathologist, and representative areas free from necrotic and hemorrhagic materials were premarked in the paraffin blocks. For each sample, a 1.5-mm core was punched from the donor blocks, and transferred to the recipient paraffin block at defined array positions using a tissue microarray instrument. Several serial sections (4 μm in thickness) were cut from all TMAs, one of which was stained with hematoxylin-eosin as reference.

### Immunohistochemistry (IHC) and scoring

Paraffin blocks that contained sufficient formalin-fixed tumor specimens were serially sectioned at 4 μm and mounted on silane-coated slides for IHC analysis. The sections were deparaffinized with dimethylbenzene and rehydrated through an ethanol gradient, including 100, 95, 85 and 75% ethanol. Antigen retrieval was performed with 0.01 mol/L sodium citrate buffer (pH 6.0) in an autoclave at 121 °C for 2 min, and endogenous peroxidase was blocked by incubation with 3% hydrogen peroxide for 10 min at room temperature. The slides were then washed in phosphate-buffered saline (PBS), blocked with 10% goat serum (ZhongShan Biotechnology, China) for 30 min and incubated with MCM6 antibodies (ab4458 Abcam, UK) and CDK5RAP3 antibodies (ab168353 Abcam) in a humidified chamber at 4 °C overnight. Following three washes in PBS, the sections were incubated with HRP-conjugated secondary antibodies (ZhongShan Biotechnology) for 30 min at room temperature. Next, the signal was developed with a diaminobenzidine (DAB) solution (ZhongShan Biotechnology), and all the slides were counterstained with 20% hematoxylin. Last, the slides were dehydrated and mounted with cover slips. For negative controls, the primary antibody diluent was used instead of the primary antibody. The staining intensity was scored as 0 to 3. The heterogeneity of staining was scored as 0 to 3, depending on the percentage of tumor cells that were positively stained. To obtain an IHC score that considered the IHC signal intensity and the frequency of positive cells, we generated a composite expression score (CES) ranging from 0 to 9. A CES of 0, 1, 2 and 3 was defined as low expression, but a CES of 4, 6 and 9 was defined as high expression.

### Western Blotting Analysis

Cells were plated into 60-mm dishes and cultured to 80% confluence. The cells were then scraped and lysed in RIPA buffer and the lysates were centrifuged at 10,000 g (4°C for 10 min). Protein concentrations were determined using the BCA Protein Assay Kit (Thermo Fisher Scientific, New York, USA) according to the manufacturer's instructions. A total of 40 µg protein from each sample was denatured and loaded into each well, separated by SDS-PAGE, and transferred to a polyvinylidene difluoride membrane (Millipore, Billerica, MA). The membranes were blocked with 5% nonfat milk at room temperature for one hour and incubated overnight with primary antibodies in PBST (1:1000). After washing with PBST, the membranes were further incubated for 1 h at room temperature with the corresponding horseradish peroxidase-conjugated secondary antibody in appropriate dilution and then washed three times with the same buffer. The membranes were detected using enhanced chemiluminescence (Amersham Corporation, Arlington Heights, IL, USA). Anti-MCM6 (ab4458), anti-CDK5RAP3 (ab157203) and anti-GAPDH (ab181602) antibodies were purchased from Abcam. Horseradish peroxidase-conjugated goat anti-rabbit IgG (A4914), and anti-mouse IgG (A0168) were purchased from Sigma. MCM6 RNA (#5915) and control RNA (#6568) were purchased from Genechem Co. Ltd. (China). MCM6-siRNA and control RNA were purchased from Genepharma Co. Ltd. We corrected the loading error according to loading controls and made comparisons between the expression levels of target proteins in tumor and normal tissue. The protein expression in the tumor was defined as high when the expression was higher than that in normal tissue, and expression was defined as low when the expression was lower than that in normal tissue.

### Immunoprecipitation

Cells were washed with ice-cold PBS and lysed in Tris-buffered saline (pH 7.4) containing 50 mmol/L Tris, 150 mmol/L NaCl, 1% nonidet P-40, 1 mmol/L EDTA, 1 mmol/L Na3VO4, 10 mmol/L NaF, 2.5 mg/mL aprotinin and leupeptin, 1 mmol/L -glycerophosphate and 4-(2-aminoethyl) benzenesulfonyl fluoride hydrochloride, and 10 mmol/L iodoacetate. Lysates were incubated on ice for 15 min before cellular debris and nuclei were removed by centrifugation at 10,000g for 5 min. Cell lysates were incubated with the MCM6 antibody (ab201683) and CDK5RAP3 antibody (ab157203) overnight at 4°C. Protein A-Sepharose (Amersham Biosciences, Piscataway, NJ) beads in a 50:50 mixture in 50 mmol/L Tris buffer, pH 7.0, were added and further incubated for another 4 h at 4°C. The immunoprecipitates were washed 4 times in Tris-buffered saline and boiled for 5 minutes in 40 L Laemmli buffer containing 0.02% blue bromophenol and 2% mercaptoethanol.

### Cell culture

Human gastric cancer cell lines, HGC-27 and AGS, were obtained from the Cell Line Bank of the Chinese Academy of Sciences. All the cell lines were confirmed free of mycoplasma contamination by PCR and culture methods. The species origin was confirmed with PCR. The identity of the cell line was authenticated with short tandem repeat (STR) profiling. These cell lines were cultured in RPMI 1640 for HGC-27 cells (Gibco, Grand Island, NY) and DMEM/F12 for AGS cells (Gibco, Grand Island, NY) supplemented with 10% fetal bovine serum (FBS) (Gibco, Grand Island, NY) and incubated at 37°C in a humidified atmosphere containing 5% CO_2_.

### Establishment of cell lines

The cell lines were established as previously described 4.

### Cell proliferation

After the indicated treatments, gastric cancer cells in the logarithmic growth phase were seeded in 96-well plates in triplicate at densities of 1×10^3^ cells per well. Cell proliferation was examined at 0, 1, 2, 3 and 4 days using the CCK8 assay. In brief, 10 μl of CCK8 in RPMI 1640 (100 μl) was added to each well, and the cells were incubated for 2 h at 37°C. Then, the optical density values were measured at 450 nm using a microplate reader (Bio-Rad, Hercules, CA, USA). In cell counting assay, stable gastric cancer cells were seeded in 24-well plates in triplicate at a density of 1×10^4^ cells per well. Cells were counted at 0, 1, 2, 3 and 4 days using the automated cell counter (Counts star, China). Statistical results were obtained from three independent experiments.

For colony formation assays, gastric cancer cells were seeded into 6-well plates at 1×10³cells/well and cultured in media supplemented with 10% FBS for 12 days. Media was changed every other day. Cell growth was stopped after 12 days in culture by removing the medium and adding 0.5% crystal violet solution in 20% methanol. After staining for 10 minutes, the fixed cells were washed with PBS and photographed. Statistical results were obtained from three independent experiments.

### Cell migration assays

Transwell chambers (polycarbonate filters of 8 μm porosity, BD Bioscience) were used in this test. The bottom chamber was filled with culture medium containing 10% FBS and the upper chamber was just filled with culture medium. 6×10^4^ stable gastric cancer cells were suspended in serum-free medium and plated in the upper chamber. After incubation for 24 h, the cells were removed from the upper chamber using a cotton swab. Cells penetrated and attached to the bottom of the filter were fixed with 4% formal dehyde in PBS, followed by staining with 0.5% crystal violet for 20 min. Next, imaging was performed under a 20×objective, and photographs were taken. The statistical results of cell numbers per image field were obtained from three independent that were averaged from five image fields.

### Immunofluorescence assay

Cells grown on coverslips were rinsed with phosphate buffered saline (PBS) and fixed with cold 4% paraformaldehyde for 5 min at RT. Subsequently, the cells were blocked with Triton X-100 at a concentration of 0.2% for 30 min. Cells were then blocked for 1 h with 5% BSA and washed for 30 min, followed by incubation with primary monoclonal antibodies against MCM6 (1:50; Abcam, Cambridge, UK), CDK5RAP3 (1:50, Santa Crus, America) overnight at 4 °C. The next day, the coverslips were incubated for 1 h in a dark room with Alexa Fluor 647 goat anti-rabbit IgG, Alexa Fluor 493 goat anti-rabbit IgG and Alexa Fluor 596 goat anti-mouse IgG (1:100dilution; Abcam, Cambridge, UK). Furthermore, the coverslips were stained with DAPI for 5 min at 4 °C. Finally, a laser scanning confocal microscope (Leica, Germany) was used to observe the expression in cells.

### Nuclear Protein Extraction

The assays were performed according to the manufacturer's instructions for the Qproteome Cell Compartment Kit (Qiagen, Germany). A total of 40 µg protein from each fraction was denatured and loaded into each well, and SDS-PAGE and Western blotting were conducted as described above. Nuclear extracts were immediately used for western blotting.

### Statistical analysis

All statistical analyses were performed using SPSS 18.0 for Windows (SPSS, Chicago, IL) and Prism 5.0 software (GraphPad). The chi-squared test was used to evaluate the difference in proportions, and Student's t-tests were used to evaluate continuous variables. Univariate survival analysis was performed using the Kaplan-Meier method, and the curves were compared using the log-rank test. Multivariate analysis was performed using the Cox proportional hazards model in an effort to further evaluate all the significant prognostic factors that were found in the univariate analysis. P < 0.05 was considered statistically significant and all P values were two-sided.

## Results

### CDK5RAP3 interacted with MCM6

**Figure 1:**
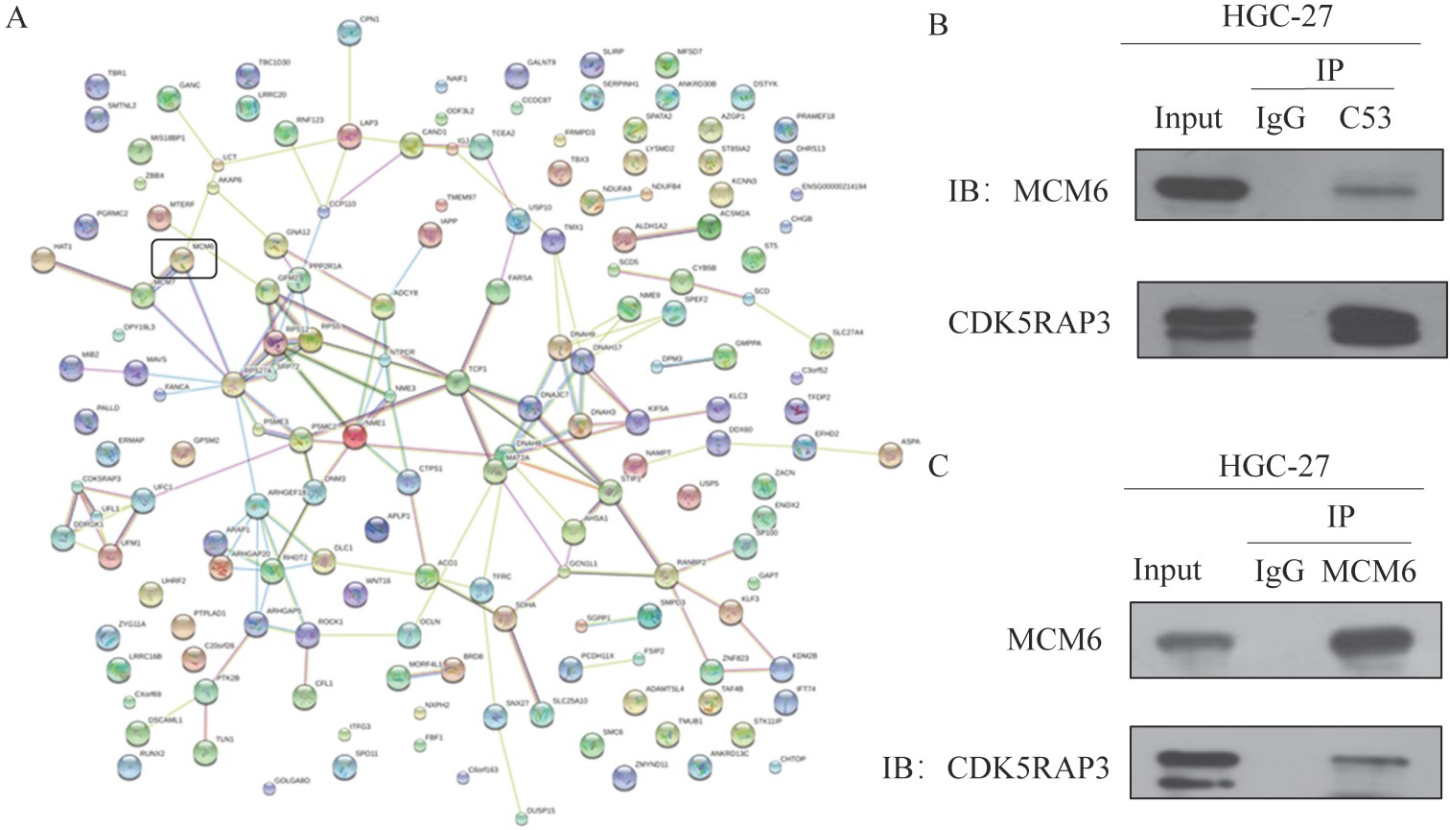
** CDK5RAP3 interacts with MCM6 in HGC-27 cells.** (A) MS results showed CDK5RAP3 is closely related with MCM6. (B) Western blot showed that CDK5RAP3 can be immunoprecipitated with MCM6 in HGC-27 cells. (C) Western blot showed that MCM6 can be immunoprecipitated with CDK5RAP3 in HGC-27 cells.

To find CDK5RAP3-associated complexes, mass spectrometry (MS) was performed. MS showed that CDK5RAP3 was strongly associated with MCM6 (Fig. 1A). To further confirm that CDK5RAP3 can interact with MCM6, an immunoprecipitation assay was performed. HGC-27 and 293T cells overexpressing CDK5RAP3 and MCM6 were incubated with antibodies against CDK5RAP3 or MCM6. Western blotting showed that CDK5RAP3 can be immunoprecipitated with MCM6 in HGC-27 cells (Fig. 1B) and 293T cells (Supplemental Fig. S1A). On the other hand, MCM6 can immunoprecipitated with CDK5RAP3 in HGC-27 cells (Fig. 1C) and 293T cells (Supplemental Fig. S1B). These results suggested that CDK5RAP3 interacted with MCM6 in HGC-27 and 293T cells.

### The effect of downregulated CDK5RAP3 was abrogated by silencing MCM6 in AGS and HGC-27 cells

To study the relationship between CDK5RAP3 and MCM6, AGS and HGC-27 cells in which CDK5RAP3 was stably knocked down were created. Changes in CDK5RAP3 expression were confirmed using Western blotting (Supplemental Fig. S2A). Additionally, the effect of MCM6 small interfering RNA (siRNA) was proven by Western blotting (Supplemental Fig. S2B). Then, the proliferative ability of these stable cells with siRNA was compared. Silencing MCM6 completely reversed the effect of the CDK5RAP3 knockdown on proliferation (Fig. 3A-B), migration (Fig. 3C-E) and colony formation (Fig. 3F-H) assays. Taken together, these data indicate that CDK5RAP3 acts as a tumor suppressor by preventing the effects of MCM6.

### MCM6 accumulated in nucleus when CDK5RAP3 was downregulated

**Figure 2:**
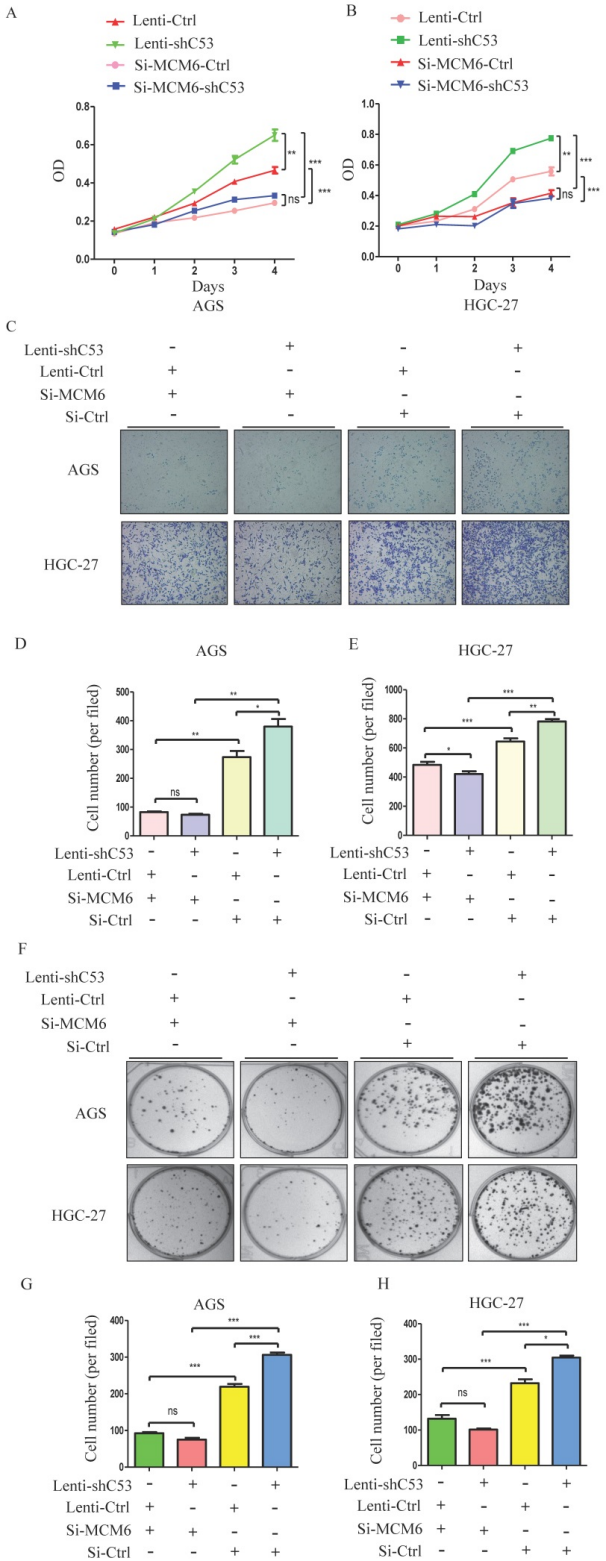
** MCM6 accumulates in the nucleus when CDK5RAP3 is downregulated.** (A) MCM6 accumulated in the nucleus in AGS and HGC-27 cells when CDK5RAP3 was downregulated when compared to the control group. (B) The expression of MCM6 was higher in the nucleus than in was in the control group via a nuclear protein extraction assay.

**Figure 3:**
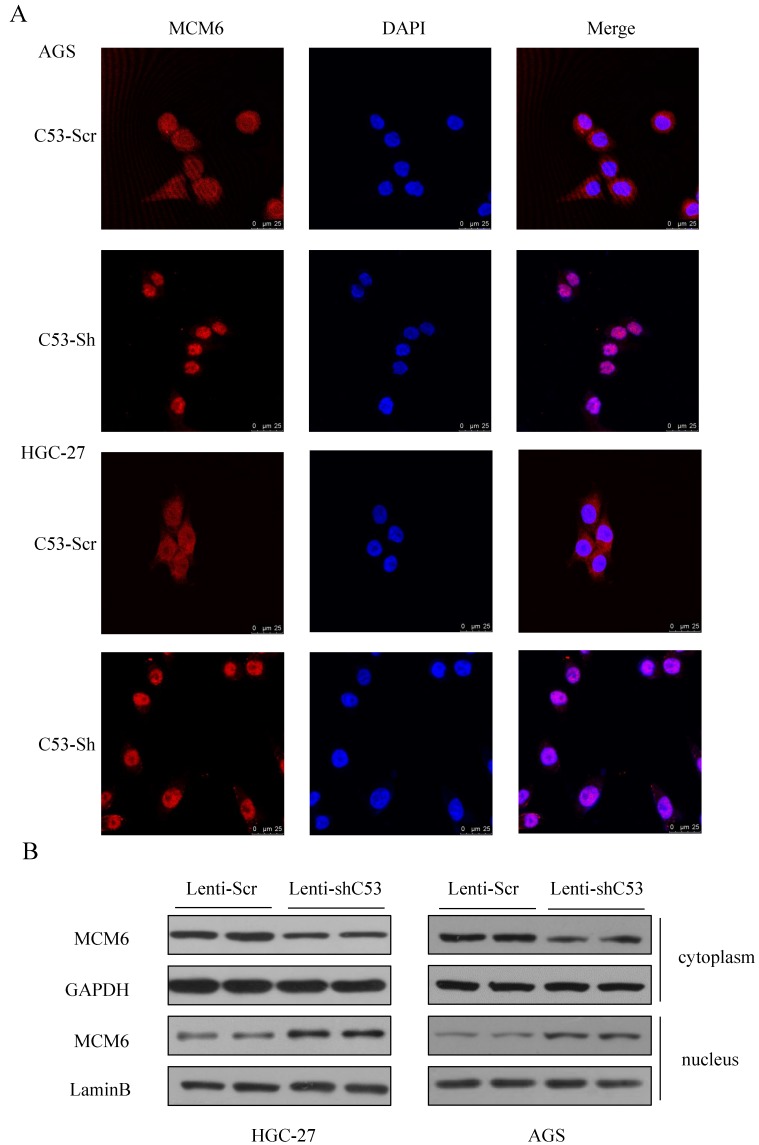
** Silenced MCM6 repressed the tumorigenicity of gastric cancer cells with stable CDK5RAP3 downregulation.** (A-B) The stimulatory effect of CDK5RAP3 downregulation on AGS and HGC-27 cell proliferation was rescued by MCM6 siRNA in CCK8 proliferation assay. (C-E) The stimulatory effect of CDK5RAP3 downregulation on AGS and HGC-27 cell migration was rescued by MCM6 siRNA. (*, P<0.05; **, P < 0.01; ***, P < 0.001; ns, no significance). (F-H) The stimulatory effect of CDK5RAP3 downregulation on AGS and HGC-27 cell colony formation was rescued by MCM6 siRNA. (*, P<0.05; ***, P < 0.001; ns, no significance).

To figure out the mechanism of the CDK5RAP3 and MCM6 interaction, two primary cell lines were introduced. Then, to confirm the changes in MCM6 when CDK5RAP3 was downregulated, an immunofluorescence assay was performed. In both established cell lines, MCM6 accumulated in the nucleus when CDK5RAP3 was downregulated (Fig. 2A). The same results were shown by a nuclear protein extraction assay (Fig. 2B). Above all, downregulating CDK5RAP3 helps MCM6 translocate into the nucleus.

### MCM6 was involved in the regulation of gastric cancer by CDK5RAP3

**Figure 4:**
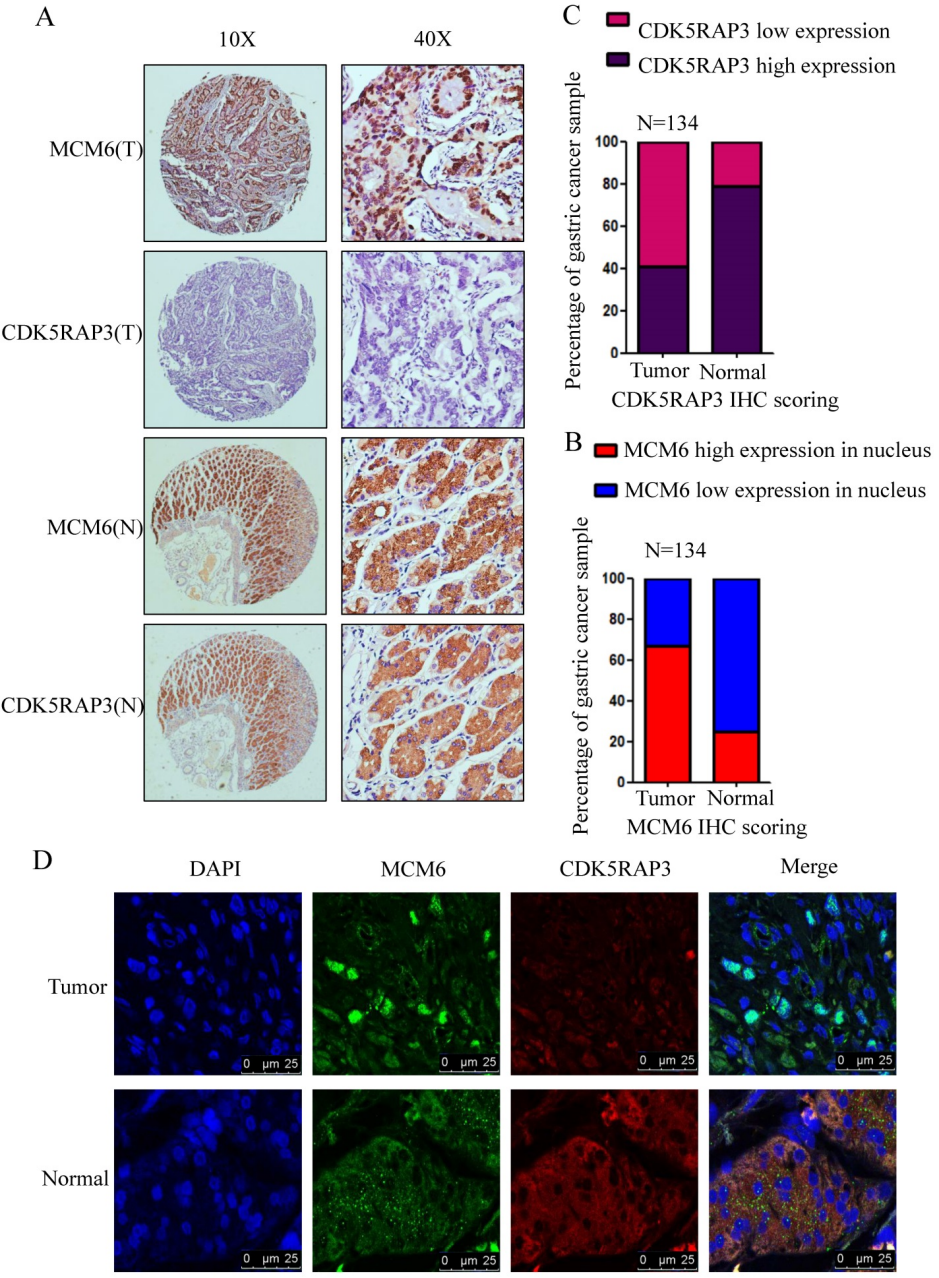
** The clinical value of CDK5RAP3 expression depends on MCM6 expression.** (A) The expression of CDK5RAP3 and MCM6 proteins in gastric tumor tissues and adjacent nontumor tissues was analyzed using IHC (representative results are shown). (B) The CDK5RAP3 expression score was higher in the nontumor tissues than in the respective tumor tissues. (C) The MCM6 expression score in the nucleus was lower in the nontumor tissues than in the respective tumor tissues. (D) The localization of MCM6 changes with the declining of CDK5RAP3 in patients' samples via immunofluorescence experiments.

To verify the association between CDK5RAP3 and MCM6, the expression of CDK5RAP3 and MCM6 proteins was detected in 134 gastric cancer samples using IHC. In gastric cancer samples, the staining of MCM6 was mostly in the nucleus, while in the adjacent nontumor tissues, the staining revealed that MCM6 remained in the cytoplasm (Fig. 4A). In these paired samples, the CDK5RAP3 expression score was significantly higher in nontumor tissues than in the respective tumor tissues (Fig. 4B). However, the total score of MCM6 expression showed no significant differences between the respective tumor tissues and nontumor tissues. Then, we evaluated the MCM6 expression score in the nucleus separately. The data indicated that the MCM6 expression score in the nucleus was significantly lower in these nontumor tissues than in the respective tumor tissues (Fig. 4C). Surprisingly, we found that MCM6 staining and CDK5RAP3 staining were exactly the same in the adjacent nontumor tissues. To further confirm this phenomenon, immunofluorescence experiments were performed. The results demonstrated that CDK5RAP3 and MCM6 colocalized in the cytoplasm in the adjacent nontumor tissues, while CDK5RAP3 had lowered expression in the tumor tissues, and MCM6 had greater nuclear expression in tumor tissues (Fig. 4D). These results demonstrate at the organizational level that the reduction of CDK5RAP3 can promote the accumulation of MCM6 in the nucleus.

### The prognostic value of CDK5RAP3 and MCM6 expression in gastric cancer

To evaluate the prognostic value of CDK5RAP3 and MCM6 expression, the Cox proportional hazards regression model was conducted (Table 1). Univariate analysis revealed that depth of invasion, lymph node metastasis, distant metastasis, and CDK5RAP3 and MCM6 expression were associated with patients' overall survival. Multivariate Cox regression analyses showed that T, or N stage, and expression of CDK5RAP3 and MCM6 remained independent prognostic factors.

**Figure 5:**
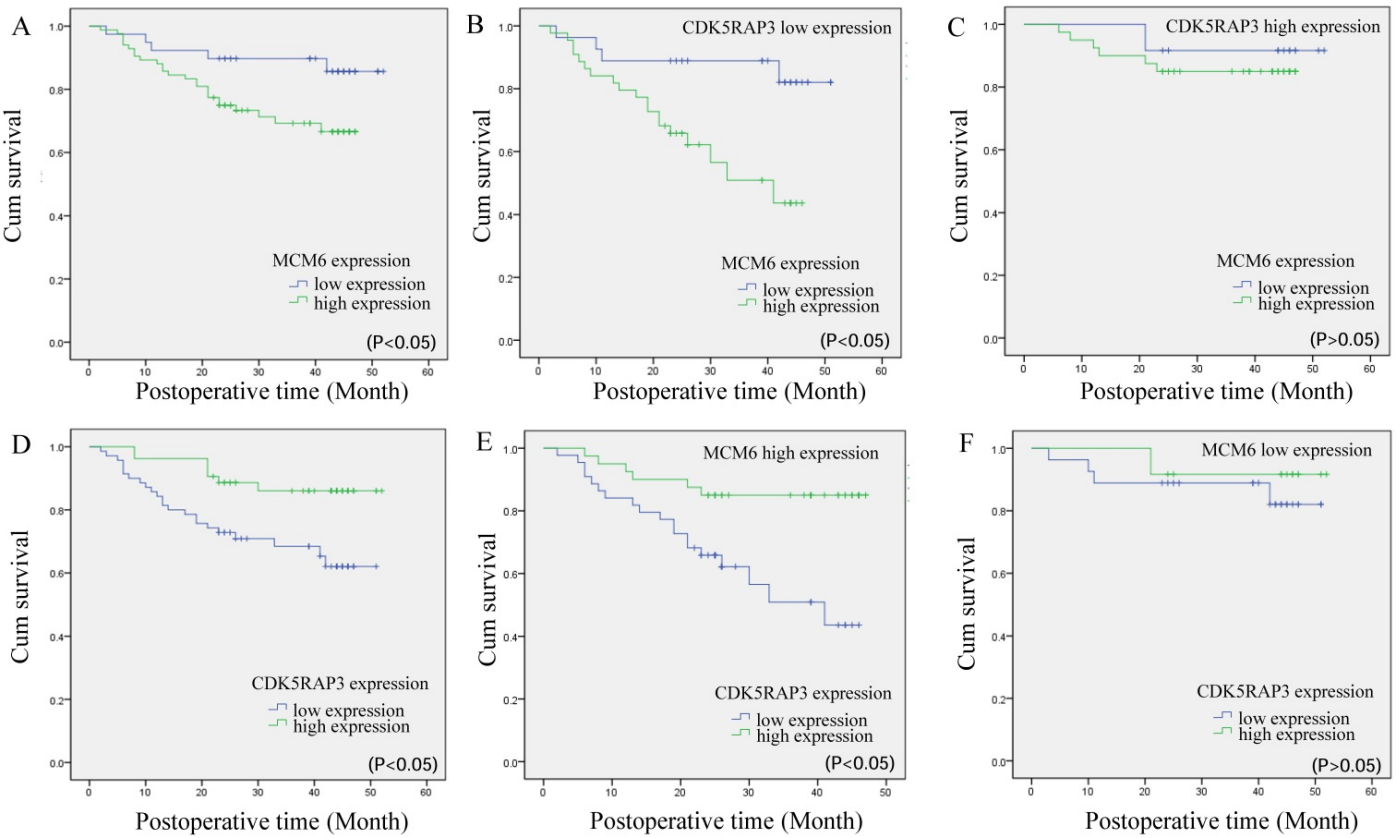
** The prognostic value of CDK5RAP3 and MCM6 expression.** (A)Kaplan-Meier survival curve of gastric cancer patients with low or high MCM6 expression (P<0.05, log-rank test). (B) Kaplan-Meier survival curve of patients with low CDK5RAP3 expression and low or high MCM6 expression (P<0.05, log-rank test). (C) Kaplan-Meier survival curve of patients with high CDK5RAP3 expression and low or high MCM6 expression (P>0.05, log-rank test). (D) Kaplan-Meier survival curve of gastric cancer patients with low or high CDK5RAP3 expression (P<0.05, log-rank test). (E) Kaplan-Meier survival curve of patients with high MCM6 expression and low or high CDK5RAP3 expression (P<0.05, log-rank test). (F) Kaplan-Meier survival curve of patients with low MCM6 expression and low or high CDK5RAP3 expression (P>0.05, log-rank test)

Next, we compared the overall survival of patients categorized by CDK5RAP3 and MCM6 expression. The overall survival rate for patients with low MCM6 expression was significantly higher than that for patients with high MCM6 expression (Fig. 5A). When CDK5RAP3 expression was low, gastric cancer patients with low expression of MCM6 had a better prognosis than did those with high CDK5RAP3 expression (P<0.05, Fig. 5B). When CDK5RAP3 expression was high, no significant overall survival difference was observed between the patients with low or high MCM6 (Fig. 5C). On the other hand, When MCM6 expression was high, gastric cancer patients with low expression of CDK5RAP3 had a poorer prognosis than did those with high CDK5RAP3 expression, while no significant overall survival difference was observed between the patients with low or high CDK5RAP3 when MCM6 expression was low (P<0.05, Fig. 5D-F). To make our findings more convincing, we selected another 70 gastric cancer samples for IHC as an independent cohort. The results fit in our findings that described above (Supplemental Fig. S3 and Supplemental Table 1). These results confirmed that CDK5RAP3 and MCM6 expression together affect clinical outcome.

## Discussion

CDK5RAP3 was first discovered as a binding partner of P35 and P39 by yeast two-hybrid assay 14. It is reported that CDK5RAP3 is expressed in various tissues and cells of the whole body, including the heart, brain, pancreas, placenta, kidney, liver, lung and skeletal muscle 15. However, abnormal expression of CDK5RAP3 is related to the occurrence and development of various malignancies. Wang et al. discovered that CDK5RAP3 acted as a tumor suppressor in head and neck squamous cell carcinomas (HNSCCs) via suppression of NF-kB activity 16.

Additionally, Zhao et al. identified CDK5RAP3 as a new candidate tumor suppressor in hepatocellular carcinoma 17. However, Mak et al. showed the opposite view, that overexpression of CDK5RAP3 promoted hepatocellular carcinoma (HCC) metastasis through p21- activated protein kinase 4 (PAK4) activation 18. The different views about CDK5RAP3 reflect the fact that the specific function of CDK5RAP3 still remains to be further interrogated.

Our primary study indicated that CDK5RAP3 expression was decreased in gastric cancer, and that cancer cell proliferation and invasion were suppressed through the inhibition of Wnt/β-catenin signaling 4. Our group also showed that CDK5RAP3 regulated the Wnt/β-catenin signaling pathway negatively by repressing AKT phosphorylation 19. Another study showed that low expression of CDK5RAP3 and DDRGK1 are related to poor prognosis in patients with gastric cancer 20. All of these studies demonstrated that CDK5RAP3 acted as an important tumor suppressor in gastric cancer. However, how CDK5RAP3 suppresses the proliferation of gastric cancer remains poorly understood. Therefore, the current study delved into a deep discussion on mechanisms underlying the suppressive role of CDK5RAP3.

To further investigate the role of CDK5RAP3 in gastric cancer, we performed MS to search for other proteins that may interact with CDK5RAP3 in HGC-27 and 293T cells. The results showed that CDK5RAP3 interacts with MCMs, especially MCM6. Some studies have illustrated that MCM6 is correlated with various proteins. For example, Chen et al. identified a direct interaction between MCM6 and P53-BP1 in human hepatoma HepG2 cells, which is essential for 53-BP1 chromatin fraction and foci formation 21. Vigouroux et al. demonstrated that Ki-67 and MCM6, both correlated with HuR, are valuable markers of poor prognosis in non-small cell lung carcinoma 22. Additionally, some studies have highlighted the clinical value of MCM6. Gauchotte et al., Helfenstein et al. and Zheng et al. showed that expression of MCM6 was strongly correlated with histologic grade and clinical outcome in patients with meningiomas, chondrosarcoma and small HCC 23-25. There are few studies investigating the role of MCM6 in gastric cancer, and the interaction of CDK5RAP3 and MCM6 had not been investigated. Therefore, studying the interaction between CDK5RAP3 and MCM6 can be meaningful in understanding the suppressive role of CDK5RAP3.

In our study, we discovered that CDK5RAP3 interacted with MCM6 and prevented MCM6 from entering the nucleus. Furthermore, knockdown of MCM6 reversed the effect of CDK5RAP3 downregulation in HGC-27 and AGS cells, which indicates that MCM6 could mediate the tumor suppressor role of CDK5RAP3 in gastric cancer. Significantly, MCM6 and CDK5RAP3 together influenced the prognostic value of patients with gastric cancer, which provided clinical support that MCM6 is involved in the tumor suppression mechanism of CDK5RAP3. However, some limitations exist in our research. We lack normal cells for control, because the normal gastric epithelial cell lines currently in use are turn immortalized that has lost the normal performance in gastric epithelial cells, particularly in proliferation. Moreover, MCM6 is associated with proliferation, so we did not use immortalized gastric epithelial cell lines for control. Besides, we evaluated the expression of MCM6 and CDK5RAP3 in patients' adjacent nontumor tissues, it also validated our findings. What's more, in our manuscript we focused on the relationship between MCM6, CDK5RAP3, clinicopathological data and prognosis of the patient, thus we just selected the most representative gastric cancer cell lines of HGC-27 and AGS to find some change in phenotype. It would be better to have more gastric cells lines to validate our findings.

In summary, our results clarified that CDK5RAP3 interacts with MCM6 and prevents MCM6 from entering the nucleus, thereby influencing the proliferation of gastric cancer, which provides a new aspect of how CDK5RAP3 may suppress tumor proliferation.

## Supplementary Material

Supplementary figures and tables.

## Figures and Tables

**Table 1:** Analysis of the Correlation Between Clinicopathological Parameters and Survival of Patients

		Univariate analysis		Multivariate analysis		
	HR	95% CI	P value	HR	95% CI	P value
Age (years)						
< 65 vs. ≥ 65	0.82	0.39-1.72	0.591			
Gender						
Male vs. Female	1.25	0.49-3.27	0.649			
Tumor size (mm)						
<50mm vs. ≥50mm	1.8	0.86-3.79	0.12			
Histology						
Well/Moderately vs. Poor	1.15	0.511-2.58	0.738			
Tumor location						
Upper vs. Middle vs. Low vs. ≥2 regions	0.79	0.56-1.11	0.17			
Depth of invasion						
pT1 vs. pT2 vs. pT3 vs. pT4	3.1	1.56-6.18	<0.001	2.18	1.12-4.24	0.022
Lymph node metastasis						
pN0 vs. pN1 vs. pN2 vs. pN3	3.03	1.69-5.43	<0.001	2.4	1.30-4,43	0.005
Distant metastasis						
pM0 vs. pM1	4.22	1.27-14.07	0.019	1.35	0.39-4.73	0.635
TNM stage						
I vs. II vs. II vs. III vs. IV	4.9	2.35-10.24	<0.001			
CDK5RAP3 expression						
Low vs. High	0.35	0.15-0.82	0.015	0.37	0.15-0.89	**0.026**
MCM6 expression						
Low vs. High	2.71	1.03-7.09	0.043	2.79	1.04-7.51	0.042
