# Supplementary material for: CDK5RAP3 Inhibits the Translocation of MCM6 to Influence the Prognosis in Gastric Cancer
Source: J Cancer. 2019 Jul 25;10(19):4488–98. doi: 10.7150/jca.32208 (PMC6746120; doi:10.7150/jca.32208)
Supplement: Supplementary file 1 — Supplementary figures and tables. [file jcav10p4488s1.pdf]

Supplemental Figure S1

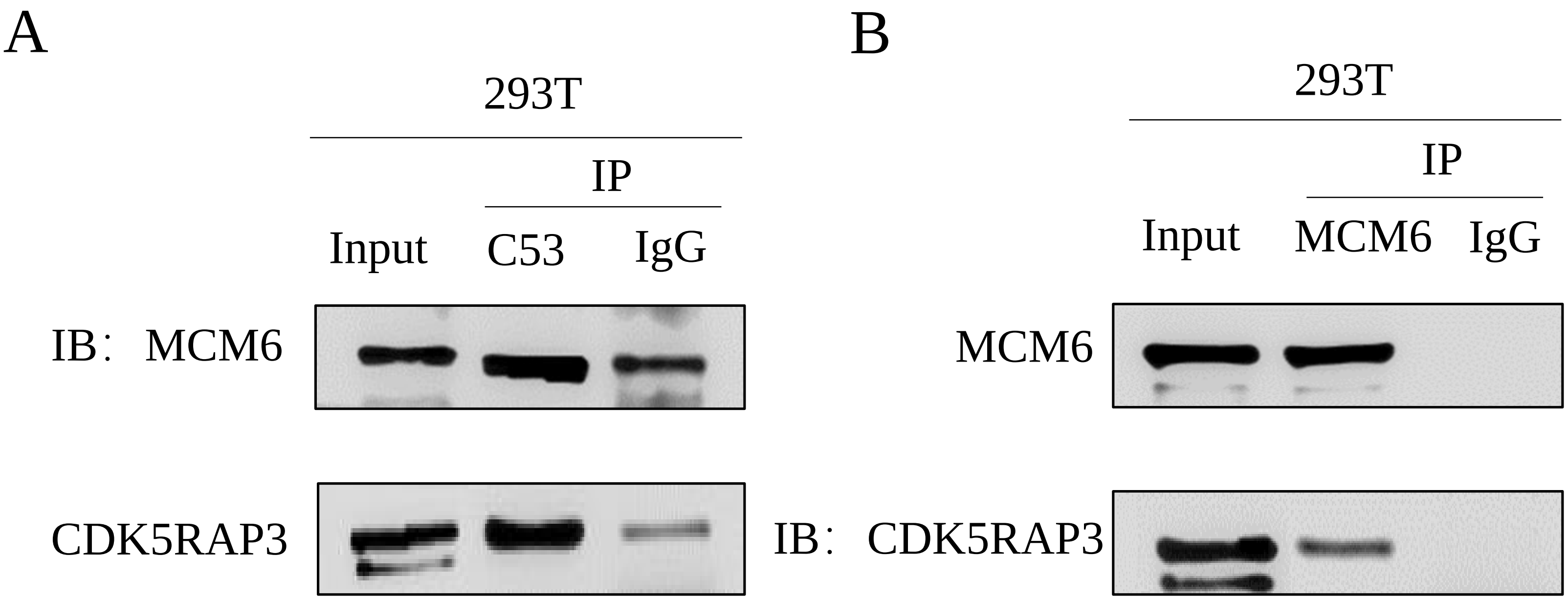

Supplemental Figure S2

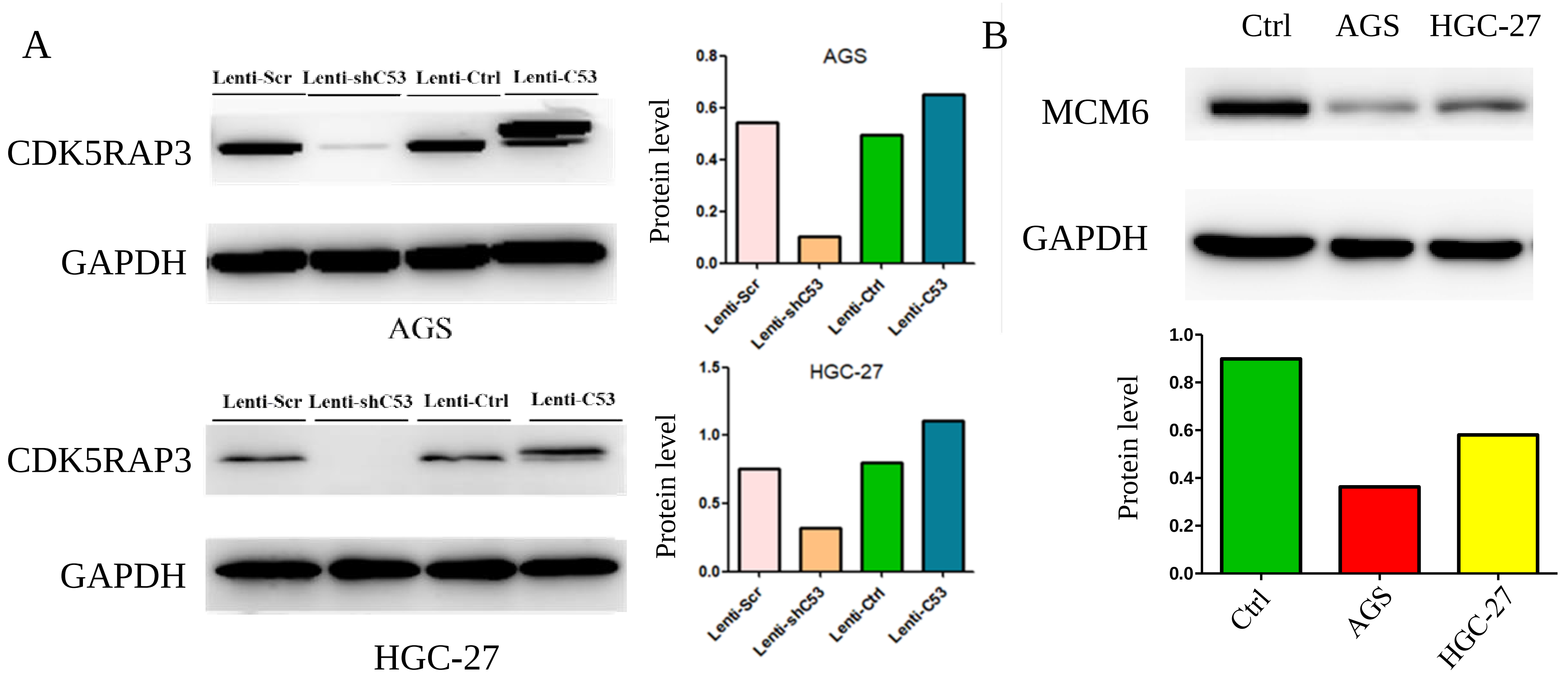

Supplemental Figure S3

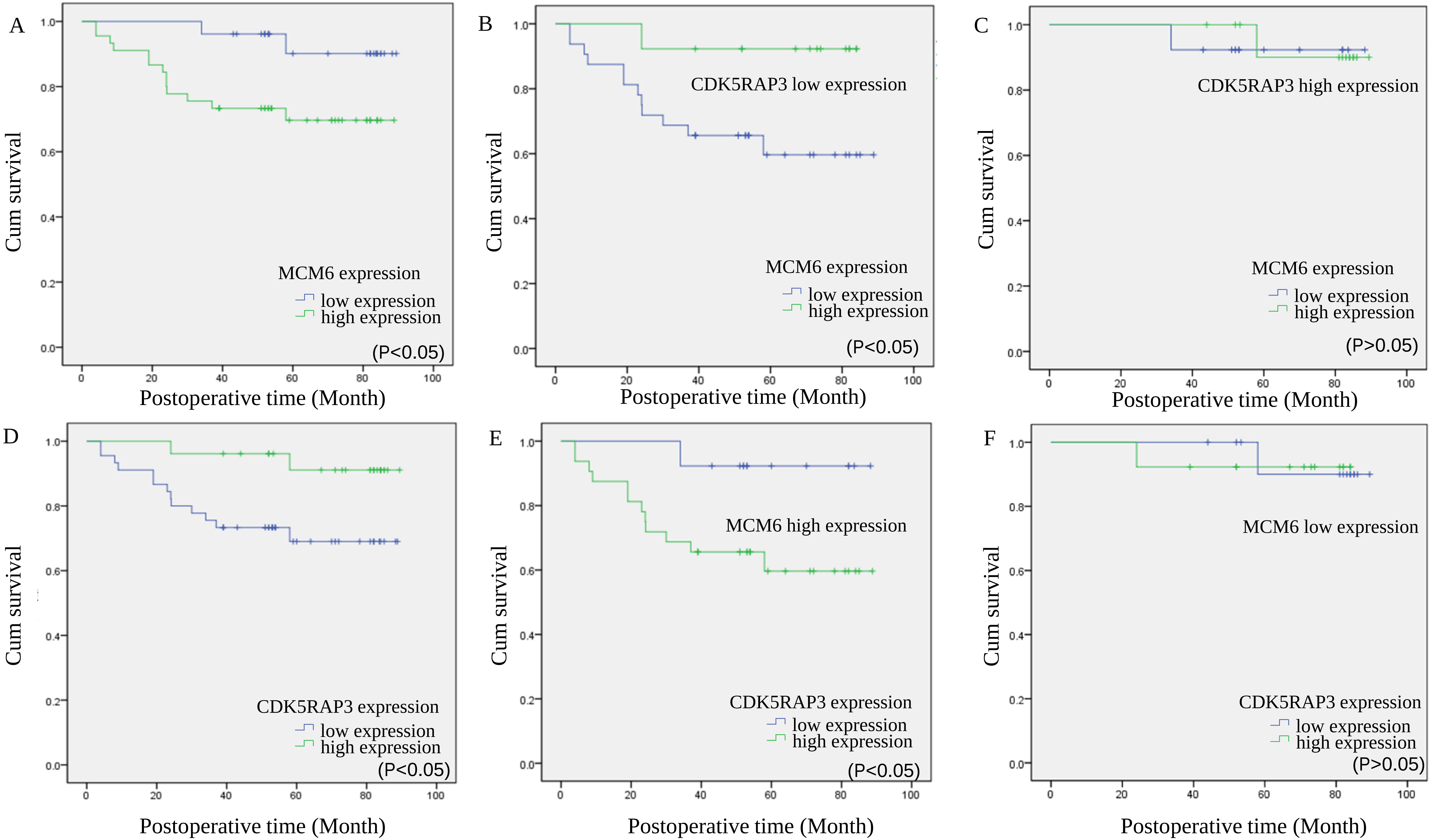

Supplemental table 1 Analysis of the Correlation Between Clinicopathological Parameters and Survival of Patients

	Univariate analysis		
	HR	95% CI	P value
Age (years)			
< 65 vs. ≥ 65	2.63	0.95-7.26	0.062
Gender			
Male vs. Female	1.03	0.29-3.64	0.966
Tumor size (mm)			
<50mm vs. ≥50mm	2.70	0.86-8.50	0.09
Histology			
Well/Moderately vs. Poor	0.25	0.03-1.92	0.183
Tumor location			
Upper vs. Middle vs. Low vs. ≥2 regions	1.18	0.70-1.99	0.527
Depth of invasion			
pT1 vs. pT2 vs. pT3 vs. pT4	3.90	1.24-12.27	0.02
Lymph node metastasis			
pN0 vs. pN1 vs. pN2 vs. pN3	5.92	2.05-17.06	0.001
Distant metastasis			
pM0 vs. pM1	6.36	1.74-23.22	0.005
CDK5RAP3 expression			
Low vs. High	0.22	0.05-0.96	0.044
MCM6 expression			
Low vs. High	4.68	1.06-20.77	0.042
